# Enhancing the Oxidation Resistance of Al_2_O_3_-SiC-C Castables via Introducing Micronized Andalusite

**DOI:** 10.3390/ma14174775

**Published:** 2021-08-24

**Authors:** Xiaoyu Wang, Saixin Wang, Yuandong Mu, Ruijie Zhao, Qingfeng Wang, Chris Parr, Guotian Ye

**Affiliations:** 1 Henan Key Laboratory of High Temperature Functional Ceramics, School of Materials Science and Engineering, Zhengzhou University, Zhengzhou 450001, China; wxysop13@163.com (X.W.); 202012192013587@gs.zzu.edu.cn (S.W.); 2 Gongyi Shunxiang Refractories Co., Ltd., Zhengzhou 450001, China; 15538387111@163.com; 3 School of Materials Science and Engineering, Luoyang Institute of Science and Technology, Luoyang 471023, China; qfwang@lit.edu.cn; 4 Imerys Aluminates, 92800 Paris, France; chris.parr@imerys.com

**Keywords:** micronized andalusite, antioxidation, Al_2_O_3_-SiC-C castables, hot modulus of rupture

## Abstract

Additions of andalusite aggregates (19 wt%) were shown in previous literature to enhance the antioxidation of Al_2_O_3_-SiC-C (ASC) castables. This work aims to investigate whether micronized andalusite has a greater influence on antioxidation improvement than andalusite aggregates. Various low contents (5 wt% and below) of micronized andalusite (≤5 μm) were introduced as a substitute for brown fused alumina in the matrix of ASC castables. The antioxidation of castable specimens was estimated by the oxidized area ratio on the fracture surface after a thermal shock test. The microstructure and phases of micronized andalusite and the castable specimens were characterized by scanning electron microscopy (SEM) and X-ray diffraction (XRD), respectively. The results suggest that the antioxidation effects of ASC castables with a low addition of micronized andalusite are effectively enhanced. The heat-induced transformation of andalusite produces SiO_2_-rich glass, favoring the sintering of the castable matrix and impeding oxygen diffusion into the castable’s interior. Therefore, the castable antioxidation is enhanced without deteriorating the hot modulus of rupture.

## 1. Introduction

Al_2_O_3_-SiC-C (ASC) castables are widely employed in blast furnace runners for their excellent thermal shock resistance and erosion resistance [1,2]. These favorable properties are mainly obtained from SiC and carbon/graphite (C) additions, which have a low wettability to molten iron and slags and provide thermal shock resistance [3,4]. However, C is vulnerable to oxidation at high temperatures, and the volatiles generated during heat treatment leads to a decrease in the compactness and strength of the castable [3,4]. Simultaneously, the decarburized layer formed after oxidation increases the castable corrosion by the slag [2]. Antioxidants are generally added to inhibit the oxidation of carbonaceous materials in castables. In this regard, the effect of various antioxidants (such as Si, B_4_C, Si_2_BC_3_N, etc.) on the oxidation resistance of ASC castables has been widely studied [5,6,7]. Although the above-mentioned antioxidants could effectively increase the antioxidation of ASC castables, they are expensive and have a high energy consumption [6,7]. However, we recently found that the partial substitution of brown fused alumina by andalusite aggregates (8–5 mm/5–3 mm/3–1 mm) could also enhance the antioxidation of ASC castables [8,9,10,11]. Andalusite is a natural mineral, which is commonly applied in refractory materials [12,13,14]. Employing andalusite could improve the volume stability and thermal shock resistance of the refractory, due to the mullitization of andalusite accompanied by a 3–5% volume expansion [8].

Based on our previous study, adopting 19 wt% andalusite aggregates could significantly promote the antioxidation of ASC castables [8,9,10,11], as the high-temperature heating prompts the transformation of andalusite into mullite and silica-rich glass. This silica-rich glass obstructs the pore channels and impedes oxygen diffusion into the castable interior [15,16,17]. Furthermore, the exuded SiO_2_-rich glass from andalusite particles reacts with fine alumina to produce secondary mullite and raise the castable densification, thereby reducing the rate of oxygen diffusion into the castable interior and enhancing the oxidation resistance [8,9,10,11].

It is suggested that the addition of micronized andalusite into the matrix may provide the enhanced oxidation resistance of the ASC castable through two mechanisms; The transformation of andalusite particles is a reaction that proceeds from the exterior to the interior, and there are more defects on the finer particle surface. Andalusite with a smaller particle size would achieve a higher conversion extent (mullitization rate) at the same heating temperature [8]. According to previous research, only 20% of the silica-rich glass phase produced by mullitization is released to the surface, and the remaining 80% remains inside [10]. Hence, reducing the particle size of andalusite aggregate is conducive to increasing the release of silica-rich glass and enhancing castable antioxidation. Conversely, most castable pore channels are distributed in the matrix, so the silica-rich glass phase produced by andalusite aggregates cannot uniformly fill the pores in the castable matrix. Moreover, there is a higher probability that the exuded silica-rich glass in the matrix will combine with the nearby alumina powder to form secondary mullite. In summary, introducing micronized andalusite into the matrix, compared with aggregates, is speculated to be a more effective route to enhancing castable oxidation resistance.

Moreover, the substitution of high-density and low-porosity brown fused alumina would deteriorate the slag corrosion resistance of the castables [18,19]. Maintaining a low content of andalusite to enhance the oxidation resistance is important for corrosion resistance. Few previous studies have researched the performance of castables containing micronized andalusite in the matrix. The current work aims to examine the influence of micronized andalusite on the antioxidation of ASC castables. The amount of micronized andalusite introduced was limited to 5 wt% or below, which was much lower than the reported andalusite aggregate content (19 wt%). Thermal shock tests coupled with the castable oxidation index were applied to evaluate the antioxidation impact [8,9]. The antioxidation mechanism was further illuminated by the phase assemblage, hot modulus of rupture (HMOR), apparent porosity (AP), residual strength ratio (RSR), cold modulus of rupture (CMOR), and castable microstructure analysis.

## 2. Experimental Procedures

### 2.1. Materials

**Figure 1:**
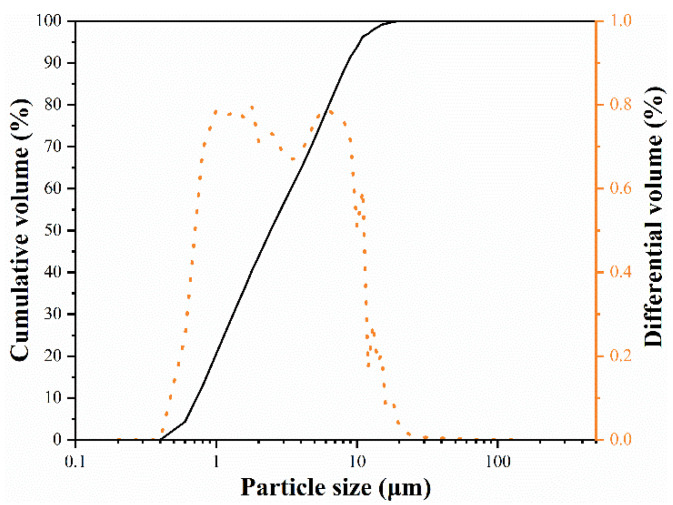
Particle size distribution of micronized andalusite.

The raw materials for the ASC castables include brown fused alumina (Al_2_O_3_ ≥ 95 wt%, ≤8 mm; Hengjia, Shandong, China), silicon carbide (SiC ≥ 97 wt%, ≤1 mm; Hengjia, Shandong, China), ball pitch (C ≥ 56 wt%, 1~0.2 mm; Hengjia, Shandong, China), Si powder (Si ≥ 98 wt%; Hengjia, Shandong, China), reactive alumina powder (CL370, Al_2_O_3_ ≥ 99.56 wt%, d_50_ = 2.14 μm; Almatis na (Qingdao) Co., Ltd., Qingdao, China), calcium aluminate cement (CAC, Secar 71, d_50_ = 13.6 μm; Imerys, Tianjin, China), aluminum powder (Al ≥ 99 wt%; Hengjia, Shandong, China), and silica fume (951U, Elkem Materials, Oslo, Norway). As presented in Table 1, the brown fused alumina was partially substituted with micronized andalusite (Durandal 59, IMERYS Co., Paris, France) in this experiment. The particle size distribution of micronized andalusite is shown in Figure 1.

### 2.2. Sample Preparation and Characterization

The ASC castable specimens were prepared according to the same procedures in the standards reported in previous work [8,9,10]. The castable specimens containing different micronized andalusite levels are denoted as M, F1, F2, F3, and F4, as listed in Table 1. The air quenching method (ΔT = 950 °C, 10 cycles) was utilized to test the fired castable thermal shock resistance. The castable specimens were held for 0.5 h in a 950 °C furnace, followed by 5 min of air cooling with a fan (air speed: 8870 m^3^/h) at room temperature. Finally, the CMOR of the fired castable bars before and after thermal shock tests were tested, and the RSR was calculated to assess the castable thermal shock resistance. Afterward, the castable specimens were cut to observe the cross-sectional oxidized layer to analyze the oxidation level after the thermal shock processes. The pixel method was used to calculate the oxidation index, which refers to the ratio of the oxidized area to the total area of the specimen section, and is used to evaluate the castable oxidation degree.

The CMOR and AP of the fired castables were obtained with a loading rate of 0.15 MPa/s, based on the Chinese Standards GB/T 3001-2007 and GB/T 2997-2015 [8,9,10]. The HMOR of the fired castables at elevated temperatures was attained via a high temperature strength tester with castables firing at 1450 °C for 3 h, according to GB/T 3002-2004 [9]. A laser particle size analyzer (HELOS & RODOS, SYMPATEC, Clausthal, Germany) was applied to detect the particle size distribution of micronized andalusite, based on the Chinese Standard GB/T 19077-2016. Scanning electron microscopy (SEM; Sigma HD, Zeiss, Oberkochen, Germany) was used to characterize the microstructures of the polished castable specimens after heat treatment at 1450 °C and of the micronized andalusite before and after heat treatment at 1450 °C for 3 h. X-ray diffraction (XRD, D8 advance, Bruker, Karlsruhe, Germany) was applied to detect the micronized andalusite phase compositions after firing at temperatures ranging from 1200 °C to 1450 °C for 3 h.

## 3. Results and Discussion

**Figure 2:**
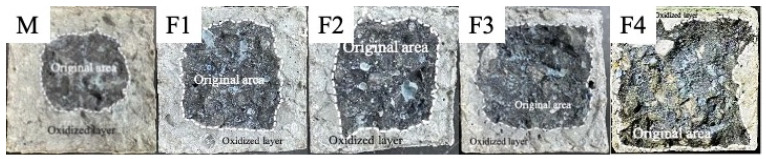
Photographs of the cross-section of the fired castables without micronized andalusite (M) and with 1 wt% (F1), 2 wt% (F2), 3 wt% (F3), and 5 wt% (F4) micronized andalusite after thermal shock tests.

**Figure 3:**
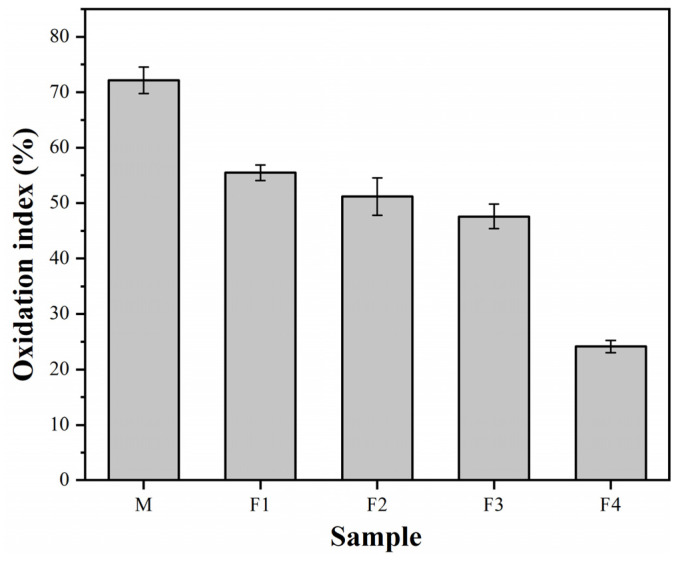
Oxidation index of the castable specimens after heat treatment at 1450 °C for 3 h.

The antioxidation effect of ASC castables is evaluated through photographs and the oxidation index (Figure 2 and Figure 3) of the calcined castable cross-sections after thermal shock tests. As shown in Figure 2, the dark regions represent the unreacted primary core, and the gray areas indicate the oxidized layer where the amount of C and SiC declines. Figure 2 also shows that the specimen M has a noticeably thicker oxidized layer than the specimen F1, and the oxidized layer thickness declines with the elevated micronized andalusite content. The oxidation index in Figure 3 quantitatively shows that the M samples have a significantly higher oxidation index than F1 samples, and the castable oxidation index gradually decreases with the increase of the micronized andalusite content. The above results denote that the antioxidation of ASC castables is noticeably improved with 1 wt% micronized andalusite and that a higher level (up to 5 wt%) of micronized andalusite leads to higher castable antioxidation.

The previous study proved that the oxidation index of the castables was not below 30% when 19 wt% andalusite aggregate (3–1 mm) was added [9]. In comparison, when only 5 wt% of micronized andalusite was applied, the castables’ oxidation index was 24.1%, illustrating that castables with a small amount of micronized andalusite achieve a comparable antioxidation effect to andalusite aggregates.

**Figure 4:**
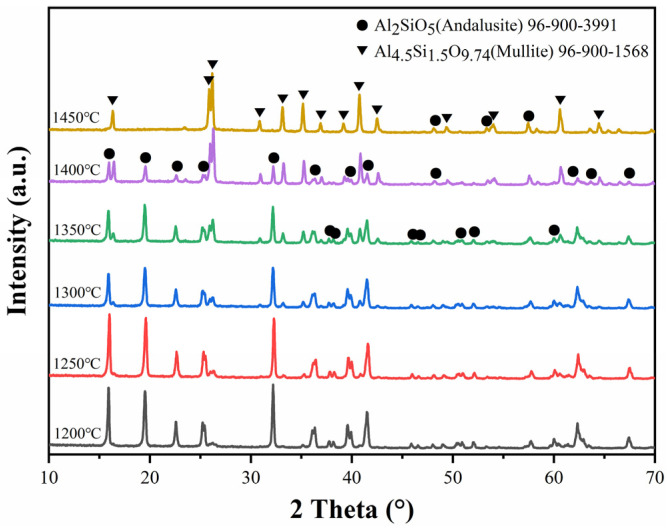
XRD patterns of micronized andalusite (≤5 μm) after firing in the temperature range of 1200 °C to 1450 °C for 3 h.

Compared with the previous study [8,9,10], since the content of the micronized andalusite in the castables of this study is low (below 5 wt%), it is hard to detect the appearance of andalusite in the XRD patterns and SEM images of the castables. Therefore, XRD patterns of pure micronized andalusite subjected to different heating procedures are illustrated in Figure 4, which visually exhibits the phase evolution of micronized andalusite during heating at high temperatures. The residual andalusite peaks gradually drop when the temperature rises from 1200 °C to 1450 °C. Meanwhile, the mullite peaks appear in specimens after calcining at 1250 °C, indicating that a portion of andalusite transforms to mullite when fired at 1250 °C. Figure 4 also signifies that the mullite peaks noticeably increase after firing at 1400 °C and that the andalusite peaks disappear after calcining at 1450 °C, demonstrating that the heat-induced decomposition of micronized andalusite is drastic at 1400 °C and completed at 1450 °C. Compared with previous research results stating that andalusite aggregates generally begin the mullitization process gradually at 1400 °C–1500 °C [10], the conversion of micronized andalusite can be completed at a lower temperature than coarse andalusite. Therefore, more silica-rich glass phases could be generated to obstruct the pore channel and increase the castable densification, thereby impeding oxygen diffusion into the castable interior. This also proves why adding low-content (5 wt%) micronized andalusite can achieve similar antioxidation properties to adding 19 wt% andalusite aggregate [9], as shown in Figure 3.

**Figure 5:**
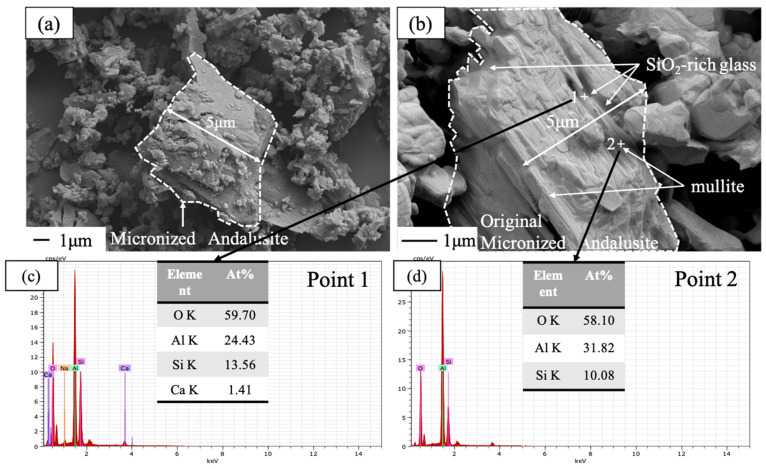
Secondary electron images of uncalcined micronized andalusite (**a**) before and (**b**) after calcining for 3 h at 1450 °C, and (**c**,**d**) the energy disperse spectroscopy (EDS) spot analyses of selected points in Figure 4b.

Figure 5a,b shows the morphology of micronized andalusite before and after heat treatment at 1450 °C for 3 h, respectively. As shown in Figure 5a,b, the degree of conversion of thermal-treated micronized andalusite is already very high when compared with uncalcined micronized andalusite. EDS spot analyses on micronized andalusite after heat treatment at 1450 °C, as shown in Figure 5c,d, detect that the particles still maintain the morphology of the original parent phase, but most of them should have transformed into mullite and SiO_2_-rich glass phase. The XRD of the andalusite after heat treatment at 1450 °C (Figure 4) also corroborates this point. The mullite produced by micronized andalusite has a short columnar shape due to its own size limitation, which is different to the mullite resulting from andalusite aggregates [15,20]. The previous study showed that a small amount of silica-rich glass phase extruded onto the surface of andalusite aggregate [9]. In contrast, as the conversion of micronized andalusite is complete after heat treatment at 1450 °C (Figure 4), the larger specific surface area and smaller particle size (Figure 1) of micronized andalusite make it easier for the silica-rich glass phase to be released to the surface, effectively bonding the surrounding particles. This further explains why adding 5 wt% micronized andalusite can achieve a similar antioxidant effect to adding 19 wt% andalusite aggregate [10]. As a result, the pore channels can be filled to a higher degree by the more extruded SiO_2_-rich glass from transformed micronized andalusite, which strongly prevents oxygen diffusion and promotes antioxidation (Figure 2).

**Figure 6:**
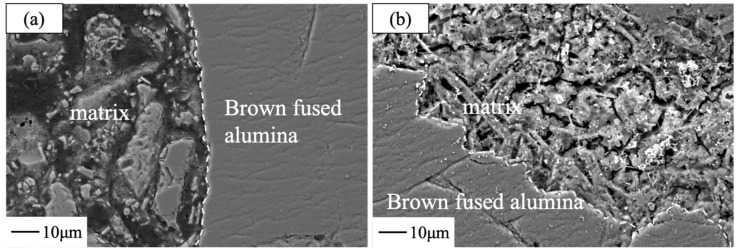
Backscattered images of the polished castable specimens (**a**) M and (**b**) F4 after calcining for 3 h at 1450 °C.

Figure 6 displays the backscattered images of the castable specimen’s oxidized layers after heat treatment at 1450 °C for 3 h. It is apparent from Figure 6a that many pores exist in the matrix, and gaps are located between the matrix and the aggregate in the M specimen, suggesting that the aggregate is not closely combined with the matrix. In comparison, the boundary between the aggregate and matrix in the F4 sample is more blurred (Figure 6b). Figure 6a,b clearly indicates that the castables containing andalusite have a much stronger aggregate-matrix interfacial transition zone. This is credited to the heat-induced transformation of andalusite which produces silica-rich glass (Figure 5) and introduces impurities, and the generated silica can partially react with alumina in the castable to produce secondary mullite during heating. Therefore, the generation and reaction of silica-rich glass creates such a strong interfacial bond. The denser connection generated by silica-rich glass and secondary mullite between the aggregate and the matrix would also block oxygen diffusion [21], thereby promoting castable antioxidation.

**Figure 7:**
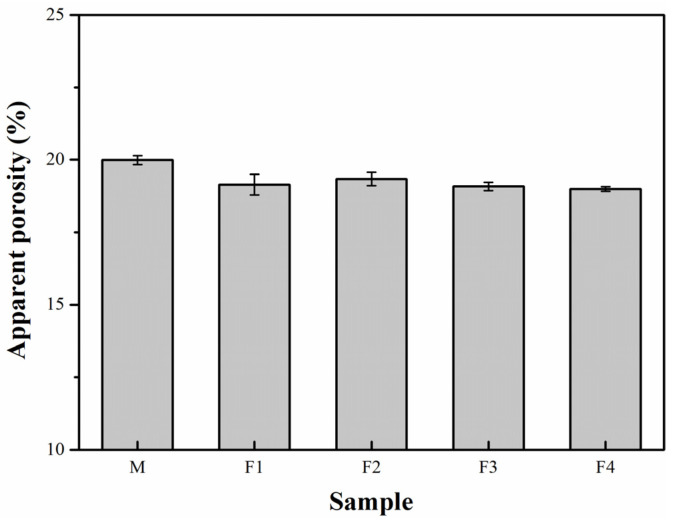
AP of the castable specimens after heat treatment at 1450 °C for 3 h.

Figure 7 implies that the AP of the castable specimens F1–F4 is slightly lower than that of the castable sample M. It should be noted that, theoretically, the formation of mullite and secondary mullite would produce a volume expansion of approximately 3% and 8–10%, respectively [2,8,15,16,17]. Due to these volume expansions, the AP in the castable should increase. In addition, the oxidation of C would also increase the porosity of samples. However, silica-rich glass (Figure 5) and the larger amount of liquid generated from the conversion of andalusite in the matrix would block the pores of castables and accelerate the sintering, consequently offsetting the volume expansion. Moreover, the improvement of oxidation resistance results in less C being oxidized in the castable, thereby reducing the porosity caused by the oxidation of C. This explains why the AP declines with an elevated micronized andalusite content. 

**Figure 8:**
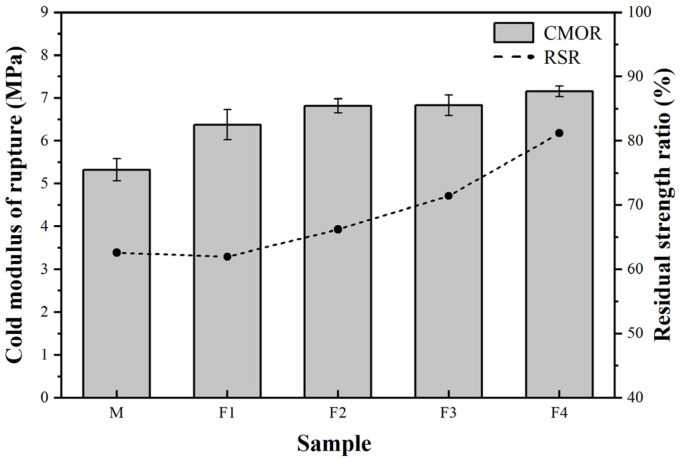
CMOR and RSR of the castable specimens after heat treatment at 1450 °C for 3 h.

The higher amount of high-temperature liquid not only benefits the decline of AP but also enhances the CMOR of the castables. Figure 8 illustrates that the CMOR of castable specimens F1–F4 is higher than specimen M, although due to the strong covalent bonding C and SiC have a low diffusivity and inhibit sintering in the oxide compositions [3,4]. The silica-rich glass and larger amount of liquid improve the castable sintering, promote the castable densification (Figure 6b), and raise the castable strength after calcining at 1450 °C.

Figure 8 also shows that the RSR of the castable specimen M is much lower than that of the castable samples F1–F4, indicating that the addition of andalusite can remarkably enhance the thermal shock resistance of the castables. The improved thermal shock resistance of castables containing micronized andalusite should mainly benefit from the silica-rich glass produced from the heat-induced transformation of andalusite, which could absorb the thermal shock stress [21,22]. As mentioned above, the improved castable antioxidation is caused by the replacement of brown fused alumina with micronized andalusite, consequently declining the oxidation degree of SiC and C. The enhanced thermal shock resistance could also be ascribed to more SiC and C remaining in the castable specimens containing micronized andalusite [3,4]. Therefore, the integration of micronized andalusite could improve the material antioxidation and enhance the castable thermal shock resistance.

**Figure 9:**
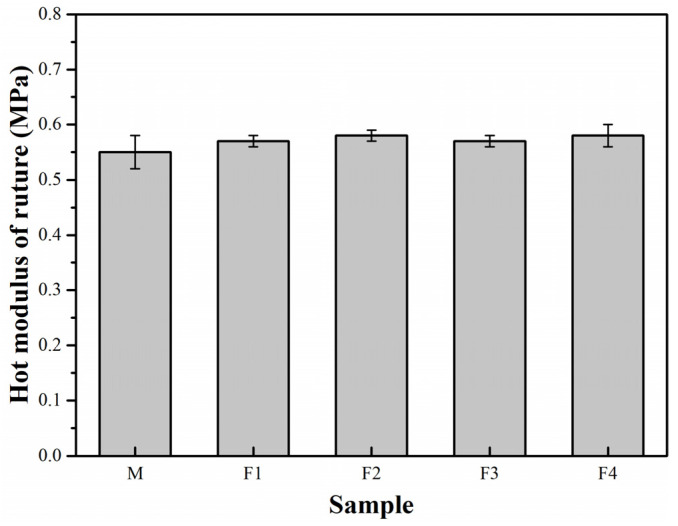
HMOR of the castable specimens after heat treatment at 1450 °C for 3 h.

As shown in Figure 9, the HMOR of the specimens F1–F4 is higher than that of the specimens without andalusite. The higher amount of secondary mullite produced from silica-rich glass (Figure 5) and alumina powder is possibly beneficial to improving the high-temperature performance of the castables containing micronized andalusite, as the secondary mullite neutralizes the negative influence of the high amount of high-temperature liquid. Moreover, the addition of micronized andalusite in excess of 5 wt% may further increase the oxidation resistance of the ASC castables, but it can be imagined that adding more micronized andalusite would produce more silica-rich glass phase, which may not benefit the HMOR of the ASC castables [23]. Further studies would be necessary to confirm the optimum addition level of micronized andalusite to ensure oxidation resistance without a reduction in HMOR and while maintaining corrosion resistance.

## 4. Conclusions

In this study, the antioxidation property of ASC castables with different micronized andalusite contents was investigated. The results suggest that the antioxidation effects of ASC castables with a low addition (5 wt% and below) of micronized andalusite are effectively enhanced. The main conclusions are drawn as follows:

The conversion ratio of micronized andalusite is higher than that of andalusite aggregates at the same heating temperature. The morphology of the fired micronized andalusite indicates that much more silica-rich glass was generated and released to the surface, which is beneficial to blocking the oxygen transition channels and hindering oxidation. This explains why a small amount of micronized andalusite addition achieves a similar antioxidation effect that a high content of andalusite aggregate does. 

Additionally, the generated glassy silica phase from micronized andalusite and the subsequent formation of secondary mullite could strengthen the bonding between the matrix and aggregate, resulting in an improved cold and hot modulus of rapture. Additionally, as more SiC and C are protected from oxidation, the thermal shock resistance of the castables is also enhanced.

## Figures and Tables

**Table 1:** Mass fractions (wt%) and particle sizes (mm) of raw materials in Al_2_O_3_-SiC-C (ASC) castables.

Raw Material	Particle Size (mm)	wt (%)				
		M	F1	F2	F3	F4
Brown fused alumina	8–5	19	19	19	19	19
	5–3	19	19	19	19	19
	3–1	19	19	19	19	19
	1–0	7.5	7.5	7.5	7.5	7.5
	≤0.074	5	4	3	2	0
Micronized andalusite	≤0.005	0	1	2	3	5
Pitch	1–0.2	3	3	3	3	3
SiC	1–0	8	8	8	8	8
	≤0.045	8	8	8	8	8
Silica fume		2	2	2	2	2
Secar 71		3	3	3	3	3
α-Al_2_O_3_ micro powder		5	5	5	5	5
Si powder		1.5	1.5	1.5	1.5	1.5
Al powder		+0.1	+0.1	+0.1	+0.1	+0.1

## Data Availability

All the data is available within the manuscript.
